# Students' acceptance of case‐based blended learning in mandatory interdisciplinary lectures for clinical medicine and veterinary public health

**DOI:** 10.1002/vro2.14

**Published:** 2021-07-22

**Authors:** Veronica Duckwitz, Lena Vogt, Claudia Hautzinger, Alexander Bartel, Sebastian Haase, Mechthild Wiegard, Marcus G. Doherr

**Affiliations:** ^1^ Department of Veterinary Medicine Institute for Veterinary Epidemiology and Biostatistics Freie Universität Berlin Germany; ^2^ Charité ‐ Universitätsmedizin Berlin Berlin Germany; ^3^ Department of Education and Psychology Department of Physics Freie Universität Berlin Germany; ^4^ Institute of Animal Welfare Animal Behaviour and Laboratory Animal Science Freie Universität Berlin Germany

**Keywords:** blended learning, case‐based learning, interdisciplinary lectures, veterinary curriculum

## Abstract

**Background:**

In German veterinary education interdisciplinary lectures (ILs) are an important and mandatory part of the curriculum as their merging character builds a useful preparation for the future profession as a veterinarian. These lectures should enable students to work on practically‐relevant and interdisciplinary cases, which should ideally be defined jointly by lecturers from different disciplines.

**Methods:**

In order to give students the opportunity to work on these cases and at the same time have contact with their lecturers and fellow students, the Faculty of Veterinary Medicine, Freie Universität Berlin, has converted its former in‐class ILs (face‐to‐face delivery format) into a blended learning format. The mandatory lectures comprise 196 curricular hours and are delivered over the course of three semesters within the veterinary curriculum. The new concept was developed over a period of three academic years and extensively evaluated (old‐new‐comparison) with regard to its acceptance and compliance with national requirements for interdisciplinary teaching.

**Results:**

A total of 306 students were asked to evaluate different aspects of the newly implemented format. Overall, more than 79% of the students attending the newly implemented blended learning format responded positively, and the evaluation showed a significant improvement of learning motivation and acceptance when compared to the traditional teaching format.

**Conclusion:**

The results indicated that blended learning is a suitable option for teaching mandatory ILs in clinical medicine and veterinary public health.

## INTRODUCTION

Interdisciplinary teaching in veterinary education is typically characterised by the collaboration of lecturers from different subjects such as pharmacology, internal medicine and animal welfare, which, in the veterinary curriculum, are often taught independently.^1^ Similar to integrated curricula that promote the merging of basic and clinical science,^2^ the intention of interdisciplinary teaching is to interlink knowledge acquired in various core subjects. Not only the comprehension of the interaction of different subjects and their mechanism, but also the application of this linked knowledge to real‐life cases is essential since this represents a preliminary stage of subsequent interdisciplinary thinking and collaboration in real‐life situations.

In Germany, the veterinary education is specified by the regulations of licensing to veterinary medicine.^3^ They include specific requirements for practically relevant and case‐based interdisciplinary lectures (ILs). For this, 196 curricular hours are allocated for an integration of clinical disciplines as well as veterinary public health issues (VPH).^3^ Additionally, the European Association of Establishments for Veterinary Education (EAEVE) recommends Day One Competences which should be considered when developing ILs. At the Faculty of Veterinary Medicine of Freie Universität Berlin (VM‐FUB), ILs were mostly offered as traditional face‐to‐face lectures (180 min) in third and fourth year (of a five‐year curriculum). The analysis of the previous ILs indicated that the emphasis was on clinical topics with only a small number of VPH topics, less than half of the lectures were interdisciplinary and only about 10 % followed a case‐based approach. Content was not coordinated between lecturers and therefore partly duplicated. Also, a student survey conducted in 2014 highlighted a low student satisfaction with the ILs presentation and choice of topics.^4^

In consequence, additional evidence was assembled how to best improve ILs to be better accepted by students, and whether a transfer in a new delivery format could be useful to address both national and international requirements for interdisciplinary teaching. The aim of the new course concept was that students are able to actively solve the interdisciplinary cases required by the TappV and at the same time, in‐person contact with fellow students and lecturers should be maintained. In order to meet all requirements, the course was changed to a Blended Learning (BL) format. Rovai and Jordan stated that BL is a flexible course design where online elements can be offered without completely losing face‐to‐face contacts; thus making it a robust educational tool.^5^ In veterinary education, BL has been successfully integrated and well accepted by students,^6^, ^7^, ^8^, ^9^, ^10^ and a survey in 2010 already pointed out a good acceptance of case‐based e‐learning.^11^ However, there is only one study analysing the acceptance of an interdisciplinary case‐based BL course in veterinary education.^1^ This collaborative pilot project ‘Neuroimmunology’ created an elective course for students with promising evaluation results, but was only conducted with a small number of students over three weeks and with three cases.

One specific requirement of ILs is training based on real life cases. This can be very well addressed by case‐based learning (CBL) which can be well combined with the BL format.^12^ CBL ‘is [a] learning and teaching approach that aims to prepare students for clinical practice, through the use of authentic clinical cases’.^13^

Furthermore, these authors state that CBL supports ‘the transfer of learning from theory into clinical application’ and may take place in small face‐to‐face groups or likewise online which ‘can work well providing attention is paid to the online learning environment’.^13^

Unfortunately, up to now there is only limited literature on best‐practice examples of how to prepare or teach ILs and whether new delivery formats such as BL or CBL can be successfully implemented in interdisciplinary clinical and VPH teaching.

The aim of our study was to analyse if it is possible to transfer an entire mandatory interdisciplinary course of 196 h over three semesters into a BL course by creating and implementing virtual clinical patients and online cases in VPH and whether there is a better acceptance of the new BL format in comparison to the old lecture‐based format. Special attention was paid to the acceptance of the mandatory BL delivery format by students and the applicability of this concept to the two interdisciplinary veterinary fields of clinical medicine and VPH.

## MATERIALS AND METHODS

### Conceptual design

Alongside the wide range of demanded topics in clinical medicine and VPH and the high number of lecture hours the following elements were identified from the national requirements (TAppV) to be the key characteristics (KCs) of the overall course under consideration: interdisciplinarity, a case‐based approach and thematic relevance for veterinary practice.

To implement the predefined wide range of interdisciplinary topics such as internal medicine, animal reproduction, food safety and outbreak investigations as well as to enhance the CBL, BL was chosen as the appropriate teaching format. Furthermore, BL offers the students the flexibility in time and place to individually work on a case on the one hand and offers the necessary support to complete the presented tasks on the other hand. The integrated virtual cases provide an opportunity for each single student to deal with the presented problem individually and at the same time to take part in an interactive process according to the respective capacities or time budget.

### Blended learning

The BL format was constructed to offer a coherent interdisciplinary case‐based content over three semesters (6, 7 and 8) for a cohort of approximately 170 veterinary students per semester. At the beginning of the 6th semester students were introduced to the course concept and the online learning environment. Cases with similar topics were arranged in combined modules, with an average number of three online cases and one subsequent in‐class lecture per module. At all times students were able to contact the responsible lecturers or technical support. During the in‐class (face‐to‐face) lectures, the focus was set on providing a forum for questions and case discussions. In some modules, additional cases or problems were offered and jointly worked through. Lecturers were encouraged to use an interactive format with emphasis on discussions and to include activating elements such as voting tools during the in‐class lecture to increase students’ participation.

This BL format differs from the known flipped classroom model as not only the background information but also the application of the acquired knowledge takes place online and at home prior to the in‐class activity.

### Selection of tool/online learning environment

As online learning environment, the web‐based authoring and learning tool tet.folio was chosen (Appendix S1). It is a web application developed at FUB, Department of Physics, Physics Education Unit.^14^ With the emphasis to develop customised interactive content, students can work page by page through the online course‐book. All individual activities like answers or notes are automatically saved by the system.^15^ Tet.folio was already used on a smaller scale at the VM‐FUB, and it is still under active development. Therefore, it was possible to integrate project specific requirements.

To offer a singular platform already familiar to the students and to facilitate access by using the already existing user account, it was decided to link tet.folio with the FUB learning management system Blackboard (Washington DC, USA). A simple landing page gives access to the content in tet.folio for predefined student groups, and the student activities in tet.folio were fed back into the user administration and grade centre of Blackboard.

### Case‐based approach

#### National and international requirements for case topics

For identification of suitable topics for the proposed case‐based approach, the project team was guided by national and international requirements. The TAppV gave broader thematic guidelines such as the field of internal medicine, reproduction and animal nutrition for companion and farm animals as well as epizootic disease control, epidemiology and food hygiene in VPH.^3^ International requirements for teaching content are specified by the EAEVE, which are more detailed but also focused on Day One Competences that a veterinary student should have at the end of their veterinary training.^16^ As the national specifications for content were defined very broadly, and the ILs can be seen as one of the major steps in preparing for real life situations, it was decided to use the EAEVE Day One Competences as supplementary guidelines (See Appendix S2).

#### Curriculum analysis for implementation of topics

The final case topics were identified with the input of internal and external experts in the field of veterinary clinical medicine and VPH. For development of two pilot online cases, two topics considered as relevant and suitable by the project team were selected.

For the implementation of the two pilot cases and for each following case, an overview of all topics taught in the clinical semesters 5–11 at VM‐FUB was compiled in order to ensure that each case is sufficiently aligned with previously taught subjects and existing knowledge.

This led to a distribution of the different cases over the three semesters of interest (6‐8) and division into different thematic modules (Appendix S3).

#### Case structure

Based on a conceptual framework for developing teaching cases, these should be relevant, realistic, engaging, challenging and instructional.^17^ With the objective to meet these core attributes, two different case structures had been developed since real VPH cases have a distinctly different workflow when compared to the virtual patients in the clinical context of this project.

The VPH cases cover various fields such as food hygiene, epidemiology and animal welfare. Based on the predefined structure, each VPH case was embedded in a mock story that continued throughout the case. After presenting the specific problem, students were asked to take specific roles in the process of solving the case such as the official veterinarian or epidemiologist. Based on the role, various tasks had to be performed that required previously acquired knowledge, and the student was encouraged to work the case from every interdisciplinary angle necessary to solve the problem. Therefore, interactive elements and exercises such as taking food samples, running and interpreting laboratory tests, performing animal welfare visits and much more were integrated into the cases. To supplement the story, the cases were enriched with animations, pictures, audio and video sequences as well as a glossary that provided relevant background information.

The virtual patients follow the pattern of a clinical patient with the sequential steps of the assessment of medical history, clinical examination, diagnostics, differential diagnosis and a treatment plan. In addition to already existing teaching material, supporting pictures, videos, animations and interactive elements were created for and implemented in the cases. Each case was enriched with different tasks of clinical decision‐making, and a glossary with background information was provided.^15^ The entire structure was designed to create realistic scenarios to optimise the student learning experience. To achieve this, the case descriptions were rich on multimedia elements and interactive tasks.

Less experienced learners require more structured cases to prevent a cognitive overload.^18^ Also, students prefer well‐structured approaches in CBL rather than open cases.^13^ To match case complexity with the learning progress of the students over time, less challenging cases were planned for the 6th semester, and the complexity increased progressively in semesters 7 and 8. In parallel, the structure of and guidance through the cases were reduced with increasing semester.

### Evaluation process

The concept of evaluation had two main focuses: (i) to assess the implementation of the national requirements (KCs: interdisciplinarity, the case‐based approach and thematic relevance to veterinary practice) into the new course concept in comparison to the previous course concept; (ii) to analyse the acceptance of the newly implemented BL format by students. This was achieved by comparing the learning motivation and subjective knowledge gain of students in the new concept with that of the traditional teaching format and assessing the students’ evaluation of general aspects of the BL format and the online case presentation.

In order to achieve these evaluation goals, four paper‐based evaluations as well as 26 case‐focussed online evaluations were conducted, in which the students from 6th to 8th semester were asked to assess the above‐mentioned items.^19^

The survey of the implementation of the national requirements (KCs) was conducted together with the survey concerning learning motivation and the subjective knowledge gain. These questionnaires compared the previous ILs and its traditional course to the new course concept. The two paper‐based surveys (‘previous survey’ and ‘survey new’) were conducted in summer semester 2017 with the students of the 6th semester. The first survey (‘previous survey’) was answered in mid‐semester (summer 2017) until which students had only been exposed to the traditional format of ILs with mandatory in‐class lectures (180 min). The second survey (‘survey new’) had to be completed at the end of the semester, after the students had a half semester of the new ILs arranged in a BL format.

For the evaluation of the KCs, students had to answer seven items: two items related to interdisciplinarity, three items on the case‐based approach and two items on thematic relevance to veterinary practice. Furthermore, they had to answer five items related to learning motivation and subjective knowledge gain. These 12 items were presented in the same questionnaire, and for each item a six‐point Likert‐type scale ranging from 1 (‘strongly disagree’) to 6 (‘strongly agree’) was used.

Questions related to general BL aspects were only asked in the end‐of‐semester (‘survey new’). All items regarding the general BL format were evaluated on the same six‐point Likert‐type scale. To verify that the questions were read properly, the score direction of one of the items was reversed.

The survey of the online cases focused on their usability and case structure and was conducted online at the end of each online case. It was answered by students from summer semester 2017 to winter semester 2018/2019. These series of surveys consisted of five items with the above mentioned six‐point Likert‐type scale and one item regarding ‘the difficulty of the cases’ with a five‐point Likert‐type scale ranging from 1 (‘far too easy’) to 5 (‘far too difficult’). Additionally, two free‐text answers were included in which students were asked to highlight what they liked and disliked most.

The evaluation was conducted with a total of 306 students. A cohort of 157 students from semester 6 to semester 8 was followed up between the summer semesters 2017 to 2018, and another cohort of 149 students in semesters 6 and 7 was asked in summer semester 2018 and winter semester 2018/2019.

Results from a detailed evaluation of the two pilot cases have been presented elsewhere.^15^, ^20^

#### Qualitative feedback from lecturers

After implementing the first five cases in a BL format in summer 2017, an e‐mail was sent to the participating lecturers (*n* = 11) to ask them for their opinion on the implemented BL delivery format in the ILs, the usability of the tool tet.folio and the overall impression of the project itself.

### Ethics vote and animal‐patient‐related experiments

The ethics proposal and animal‐patient‐related experiments are the same as published earlier.^15^ Using animals for educational purposes is classified as an animal experiment.^21^, ^22^ The animal‐patient‐related examinations, procedures and handling protocols were approved by the legal authorities (LAGeSo, L 0001/17).^15^ The ethics proposal for the evaluation by the students was approved by the Ethics Committee of the Charité at Campus Benjamin Franklin (application number EA4/125/18).

### Statistical analysis

Data analysis was performed with R version 3.6.0 (The R Foundation for Statistical Computing, Vienna, Austria). The R package sjPlot^23^ was used for plotting diverging stake bars according to the recommendations of plotting Likert scales by Robbins and Heiberger.^24^ The surveys were implemented in the course evaluation tool Unizensus 5.4.14 ebfu (Blubbsoft GmbH, Berlin, Germany) which is routinely used for teaching evaluation at FUB. To assess the reliability of the used Likert‐type scales Cronbach's alpha was calculated with *α* > 0.6 indicating good reliability.^25^

Answers to the KCs (interdisciplinarity, case‐based approach and practical relevance) as well as the learning motivation and subjective knowledge gain were dichotomised into agreeing and disagreeing with the stated fact (Likert scale agreement ≥ 4) to evaluate the difference between the previous and the new concept. To calculate the relative acceptance ratio (RAR), a Poisson regression with a binary outcome was used. The RAR describes the factor by which the percentage of students agreeing with the stated facts increased. Additionally, 95% confidence intervals (CI) and *p*‐values were reported. A *p*‐value ≤ 0.05 was considered significant. For the overall agreement to items in the same category, a mixed model was used with a random effect for student to take the repeated measurement of the student opinion into account. The mixed regression model was calculated using the R package lme4 and visualized using the package forestplot.^26^

To explore the diminishing returns of the BL modules regarding the learning motivation and subjective knowledge gain as well as the KCs, a two generalised additive linear models were fitted using the R package mgcv.^27^ To model the average Likert‐type score depending on the number of completed BL modules, the Likert‐type score of all items in the key components and the learning motivation as the outcome were used. The number of completed BL modules was modelled using a restricted cubic spline, and a random effect for student was added for the previously stated reason.

## RESULTS

### Comparison of the previous and new concept

**FIGURE 1:**
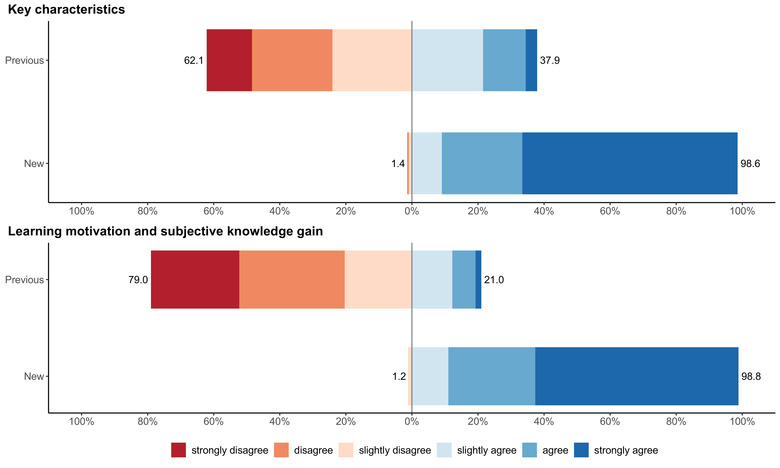
Summary of key characteristics (seven items) and learning motivation and knowledge gain (five items) of previous and new course concept of interdisciplinary lectures at the Faculty of Veterinary Medicine at Freie Universität Berlin, in summer semester 2017. Evaluated by students of 6th semester on a six‐point Likert‐type scale from 1 (‘strongly disagree’) to 6 (‘strongly agree’). Represented by a diverging bar chart with a zero line; on the right side the positive answers are shown in blue and on the left side the negative answers are shown in red

The first evaluation of the previous course concept of ILs took place in the mid.‐semester (summer 2017) with students of the 6th semester, and 109 of 157 (69.4%) students took part in this survey. The first scale regarding the KCs of ILs consists of seven items with *α* = 0.9. When combining all seven items, 37.9% of all students answered the items positively (score 4 ‘slightly agree’ to score 6 ‘strongly agree’) whereas 62.1% gave it a negative evaluation (score 1 ‘strongly disagree’ to score 3 ‘slightly disagree’). The five items of the scale for learning motivation and subjective knowledge gain had an *α* = 0.92. Most students (79.0%) evaluated the learning motivation and knowledge gain of the old lecture format negatively, while only 21% gave it a positive evaluation (Figure 1).

At the end of the 6th semester (summer 2017), 60 of 157 students (38.2%) evaluated the new course concept. Here the KC score had again a good reliability with *α* = 0.82, and more than 98% evaluated them positively. Cronbach's alpha of the learning motivation and subjective knowledge gain was *α* = 0.81, and again more than 98% of students gave a positive evaluation.

**FIGURE 2:**
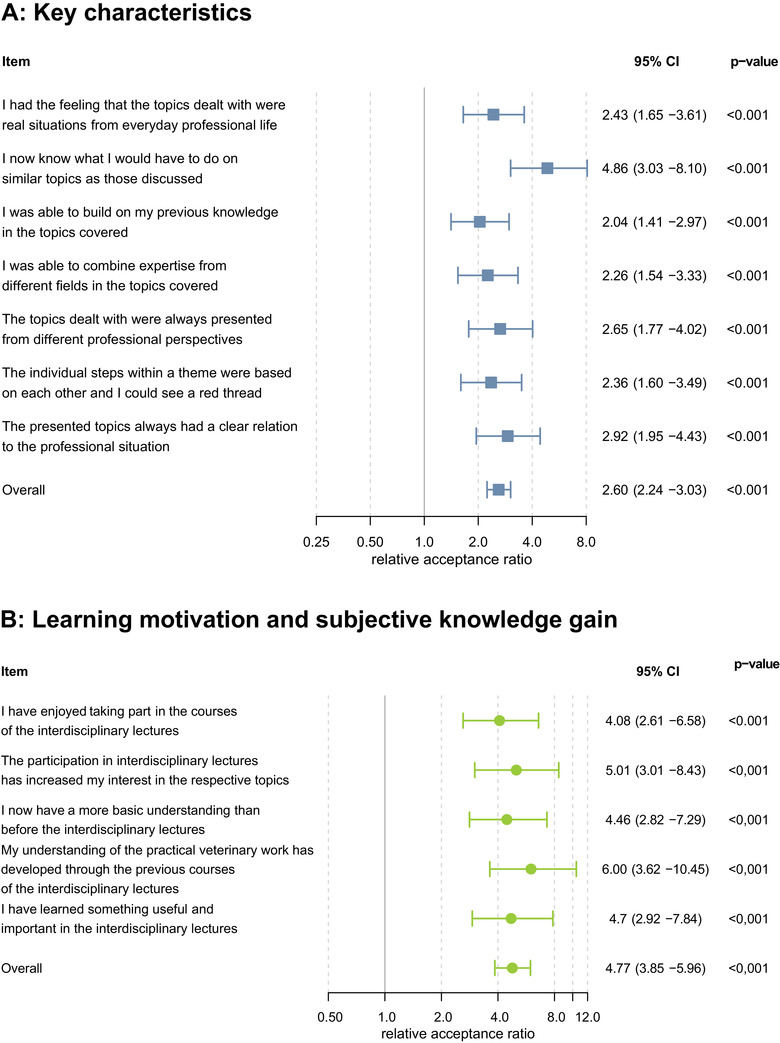
Forest plot of the key characteristics of IL (a) and learning motivation and subjective knowledge gain (b) at Faculty of Veterinary Medicine, Freie Universität Berlin. Relative acceptance ratio was measured with Poisson regression

All items were rated significantly better in the new course concept (*p* < 0.001) with the share of students evaluating the KCs positively more than doubling (RAR 2.60 [95% CI 2.24–3.03]). The learning motivation and subjective knowledge gain were evaluated positively by a proportion of students more than four times as large (4.77 [3.85–5.96]). Especially the items ‘I now know what I would have to do on similar topics as those discussed’ and ‘My understanding of the practical veterinary work has developed through the previous courses of the ILs’ has been strongly improved in the new course concept with RAR of 4.86 (3.03–8.10) and 6.00 (3.62–10.45) (Figure 2).

### The blended learning format

**FIGURE 3:**
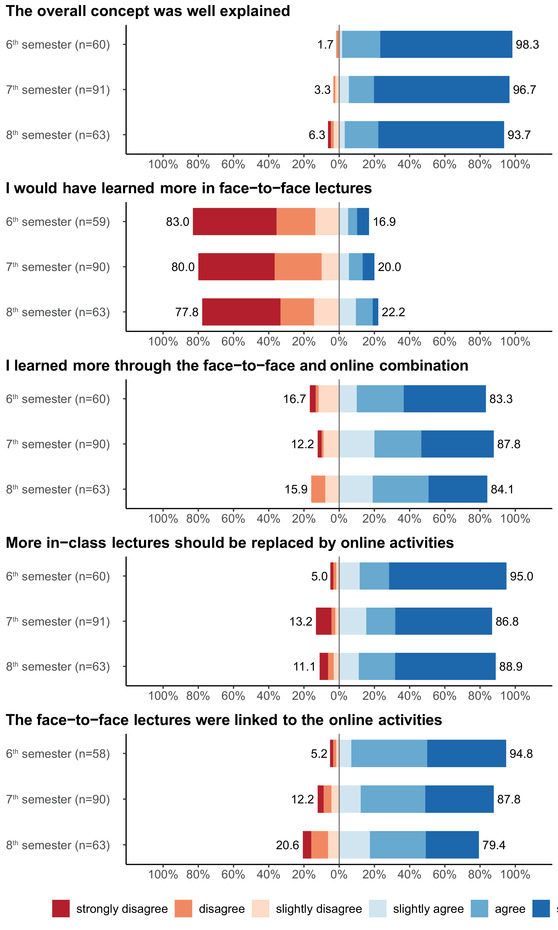
Evaluation of the blended learning format of interdisciplinary lectures at Faculty of Veterinary Medicine, Freie Universität Berlin in 6th semester 2017, 7th semester 2017/2018, 8th semester 2018. Assessment on a six‐point Likert‐type scale from 1 (‘strongly disagree’) to 6 (‘strongly agree’) shown on a diverging bar chart with zero line between negative and positive answers. For better comparison between the three semesters, all items were divided in the semesters 6, 7 and 8

The BL format of the new concept of ILs was evaluated from summer semester 2017 to summer semester 2018 following the same cohort of 157 students in 6th semester to 144 students in 8th semester. Over the sequence of three semesters, 445 students were registered in the above‐mentioned semesters (each student on average three times), and 216 (48.5%) completed their surveys on the last course day of the respective semester. The BL format was consistently rated well (Figure 3). The highest proportion of positive responses (>83%) in almost all items was seen in students of the 6th semester (first year of ILs). The same cohort in subsequent semesters 7th and 8th also rated them very positively (>79%). In the reversely coded item ‘I would have learned more in face‐to‐face lectures’, students evaluated negatively with >77% negative answers in all three semesters. In summary, all items in all three semesters were evaluated consistently positively by >79% of the students.

### Case‐based evaluation

To assess whether there is a difference between the usability and the case structure of the clinical and VPH online cases, a short evaluation after every online case took place. During the period from summer semester 2017 to winter semester 2018/2019, in total 26 online cases (12 VPH/14 clinical) were offered to the students from 6th to 8th semester during ILs. From the two different cohorts 601 students were registered for the courses in this time. In total, 1983 surveys were completed during these semesters of which 1022 were related to clinical case modules and 943 VPH cases. For 18 surveys that categorisation was missing. A total of 3846 surveys were offered to students during this period. Thus, a response rate of 51.5% was reached.

Results for the five items related to usability and case‐based structure were combined and presented for the clinical and the VPH cases. In both categories, more than 96% of students answered each of the five items positively.

### Diminishing returns of the new concept

Diminishing returns were estimated from 516 surveys in the years 2017–2019. The surveys were conducted with students from 6th to 8th semester at the end of the respective semester. Students had worked on between two and seven BL modules up to the point of the survey.

**FIGURE 4:**
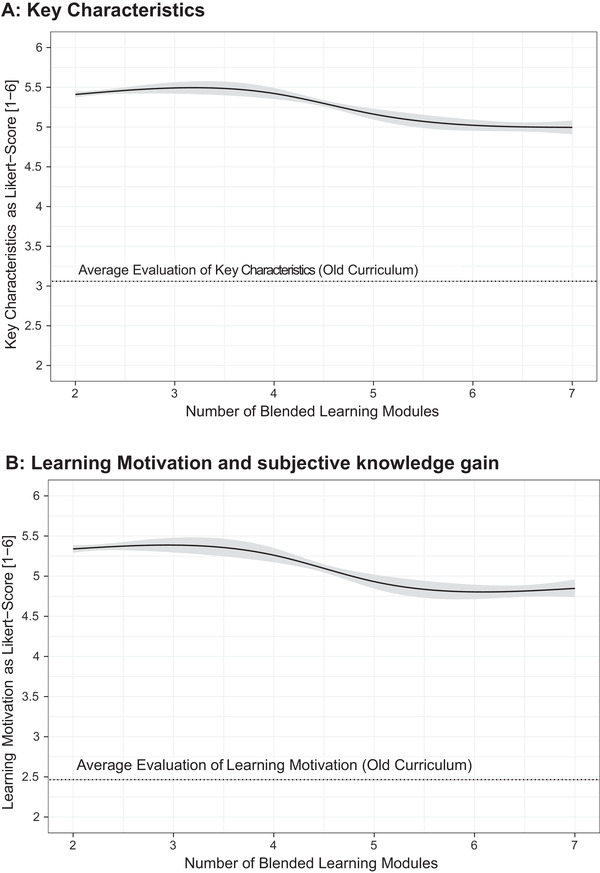
Diminishing returns of (a) key characteristics and (b) learning motivation and subjective knowledge gain of interdisciplinary lectures at Faculty of Veterinary Medicine, Freie Universität Berlin as a function of the already completed e‐learning modules. The students were surveyed after 2, 4, 5 and 7 blended learning modules. Note that 95% confidence intervals were shown as a shaded area

The mean of all items in the previous course concept regarding the KCs without any exposure to BL modules was 3.06. The arithmetic mean of all items after two BL modules (after 6th semester in 2017 and 2018) was 5.41 (95% CI 5.36–5.46) and slightly dropped to 4.99 (4.90–5.08) after seven BL modules (after 8th semester 2018) (Figure 4). Despite the decrease by 0.42 Likert points after seven modules, the BL concept was still rated 1.93 Likert points higher than the traditional course concept (Figure 4).

The learning motivation and subjective knowledge gain was rated with a mean of 2.47 in the previous course concept. After two BL modules, the average score was 5.34 (5.28–5.40) and after seven modules 4.85 (4.73–4.96). In total, the learning motivation and subjective knowledge gain decreased by 0.49 Likert‐points over the seven BL modules but still was 2.38 Likert‐points higher (after seven BL modules) when compared to the previous IL course concept.

### Qualitative feedback from lecturers

Feedback from the lecturers (response rate: 63.6%) showed that they considered BL as a useful delivery format for ILs and agreed on a good usability of tet.folio. Additionally, two lecturers expressed the wish to participate in the development of additional cases. One lecturer was not certain about maintaining the high quality of the cases once the funded project ends and another pointed out that there is a higher investment of time at the beginning but saving of time in the long term.

## DISCUSSION

In veterinary medicine ILs play an important role in education and preparation for real life situations. However, implementation of such scenarios into the curriculum so far was not satisfactory for the students and did not meet the national or international requirements for teaching in veterinary medicine. The innovation in this project is the implementation of BL into a mandatory and complex course concept with a high number of participants over a period of three curricular semesters and a high number of lecture hours in each semester. The ILs in veterinary medicine have not yet been converted into BL nor have they been evaluated for their acceptance by students to this extent.

Results show that the three KCs of the national requirements are much better implemented in the new course concept using BL compared to the traditional approach. We think that especially through the BL format it was possible to implement these KCs more successfully. The institutes and clinics involved were able to integrate their content into the collaborative online cases and coordinate the content to create a convincing and realistic case. The online multimedia format presented a platform to combine the interdisciplinary content optimally and offered a coherent case or patient for the student to work on interactively.

Looking at the learning motivation and the knowledge gain, the students were asked to evaluate subjectively, a direct relation to the improved implementation of the three KCs and the chosen case‐based, and BL format can be assumed. This not only underlines that these characteristics are important to successful interdisciplinary teaching but also that the chosen delivery format enhances student motivation and knowledge gain subjectively. The findings about increased motivation of students taking part in BL coincide with other studies.^28^, ^29^

The BL format was evaluated very positively by the students through all three semesters; this again agrees with previous studies.^6^, ^7^, ^8^, ^9^, ^10^ We think that a great impact to the successful implementation and acceptance of this delivery format was given by the predetermined order of action within the BL. The task to address the problem individually first, before meeting for interactive further discussions, presumably led to a better preparation of the course and therefore a better and more meaningful in‐class lecture with more focussed discussions and a higher value to the students. This assumption is also affirmed by the good results for learning motivation and knowledge gain and was also shown by previous studies about the combination of CBL and BL.^1^, ^11^, ^30^

The diminishing returns that we observed in our evaluations are considered minor. We conclude that the positive feedback in our continuing surveys was not solely a result of the rather poor perception of ILs prior to the start of this project in 2014,^4^ but is related to the general acceptance of the new delivery format over a sequence of seven BL modules.

In other studies, online formats are designed for one single discipline only.^31^, ^32^, ^33^ Considering the individual short surveys of the single online cases regarding the usability, we can say that despite the two different case‐structures or subject content both thematic fields of clinical medicine and VPH with all their relating issues were successfully integrated. In this project the VPH content was implemented to the same extent as the clinical content and students’ evaluations did not differ between the two veterinary fields or subject content which shows that both thematic fields can be well combined and integrated into a mutual BL format. In both areas it was possible to create a pool of different highly important cases, which enables students to work on every of these cases even if they cannot be seen during the clinical year or VPH internships.

Results show that the acceptance of this implemented BL delivery format is very high. Also, the realisation of the national requirements was achieved much better through BL. The international requirements were also addressed within this BL project. All in all, one can assume that BL is not only a relevant alternative to the traditional course concept but indeed a significant improvement.

A complicated matter especially at the beginning of the project was to encourage the lecturers of different disciplines at our faculty to collaborate and to coordinate the IL content. However, in summary qualitative feedback from lecturers was very positive. The results of the carefully planned surveys aided in convincing lecturers of the approach, and the developed modules now are a regular mandatory part of the curriculum at VM‐FUB.

Unlike in medical education where the integrated curriculum has been widely implemented, in German veterinary education the implementation of an entirely integrated curriculum has not yet taken place, and the traditional curricular structure (separation of basic science and applied science) which has already been considered insufficient in health education^2^, ^34^ is still present. However, the ILs and their presented revision are a positive step.

As limitations we would consider the lack of objective tests to analyse an improvement of learning efficiency compared to the traditional course concept in addition to the subjective surveys we took. Unfortunately, there are no tests scheduled for the ILs in Germany. To refer to subsequent tests in the following semesters would not show the influence of this modified course correctly as the parallel running courses would result in a mixing of knowledge and learning progress of all courses in the relevant semesters.

Also, the educational staff was not able to spare more time for an experimental setting and run two parallel courses to establish a comparison between the traditional and newly implemented teaching method with the same content.

The survey of the traditional course was only possible in the summer semester 2017 and not in the following semesters as it was gradually adapted to the given requirements due to selection procedures for the remaining lecture times. Therefore, only the summer semester 2017 was a representative semester for the traditional teaching format.

## CONCLUSION

This study shows a successful step‐by‐step implementation of a BL format over several semesters in the ILs, a mandatory course in the German veterinary education. By converting the complete course into online cases with associated classroom sessions, different topics could be integrated in one large coherent course concept, which was very well evaluated and accepted by the students. In contrast to the traditional course concept with face‐to‐face teaching the chosen BL format was able to implement the teaching requirements for ILs in veterinary medicine much better. Also, it was a useful delivery format to enable interdisciplinary and case‐based teaching both in clinical veterinary medicine and in VPH equally good. We hope that this study will motivate and help other universities to implement innovative delivery formats such as BL into traditional interdisciplinary courses as well as extensive courses.

## AUTHOR CONTRIBUTIONS

Lena Vogt, Veronica Duckwitz, Claudia Hautzinger and Marcus G. Doherr designed the study. Lena Vogt and Mechthild Wiegard planned and submitted the request for the animal experiments. Sebastian Haase developed the learning platform and contributed the technical support. Lena Vogt, Veronica Duckwitz, Claudia Hautzinger and Marcus G. Doherr conducted the study. Lena Vogt and Veronica Duckwitz drafted the paper. Veronica Duckwitz, Lena Vogt and Alexander Bartel analysed the data. Lena Vogt, Veronica Duckwitz, Claudia Hautzinger, Alexander Bartel, Sebastian Haase, Mechthild Wiegard and Marcus G. Doherr discussed the results and contributed to the final manuscript. Marcus G. Doherr, Lena Vogt and Veronica Duckwitz are the guarantors of the overall content.

## Supporting information

Supporting Information
