#  

**DOI:** 10.1002/vro2.21

**Published:** 2021-09-15

In this article,^1^ there was a typographical error in the footnote denoted by an asterisk: “* Joint first authorship. VD un LV contributed equally to this paper.”

The footnote should read as follows:

“* Joint first authorship. VD and LV contributed equally to this paper.”

In Figure 3, in the legend at the bottom of the figure which details the descriptors for each of the six‐points in the Likert‐type scale, the text “strongly agree” has mistakenly been cut off due to a formatting error. The corrected version of Figure 3 has been provided here:

**FIGURE 3:**
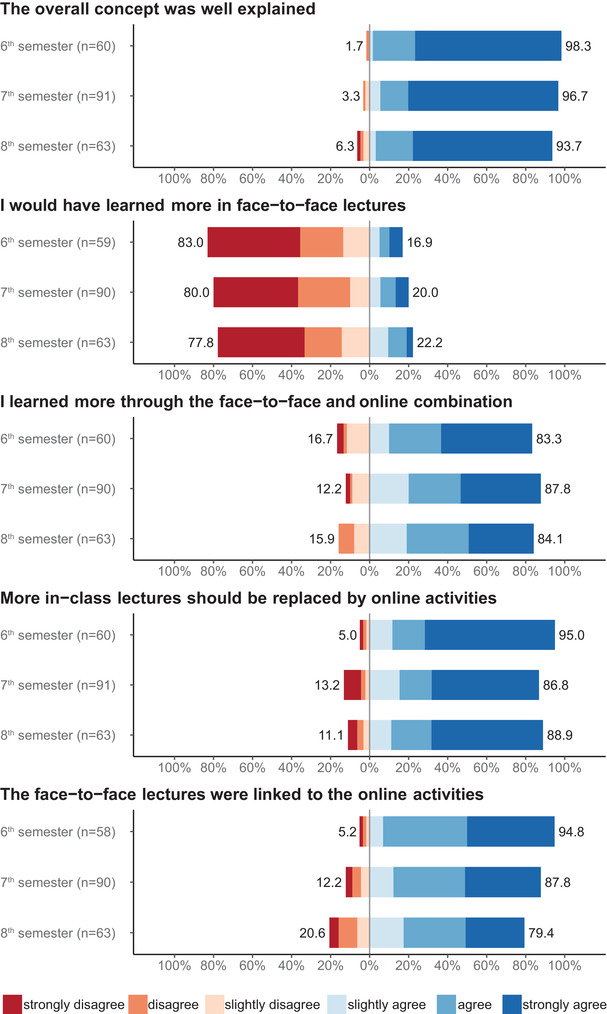
Evaluation of the blended learning format of interdisciplinary lectures at Faculty of Veterinary Medicine, Freie Universität Berlin in 6th semester 2017, 7th semester 2017/2018, 8th semester 2018. Assessment on a six‐point Likert‐type scale from 1 (‘strongly disagree’) to 6 (‘strongly agree’) shown on a diverging bar chart with zero line between negative and positive answers. For better comparison between the three semesters, all items were divided in the semesters 6, 7 and 8
