# Supplementary material for: Students' acceptance of case‐based blended learning in mandatory interdisciplinary lectures for clinical medicine and veterinary public health
Source: Vet Rec Open. 2021 Jul 22;8(1):e14. doi: 10.1002/vro2.14 (PMC8297990; doi:10.1002/vro2.14)
Supplement: Supplementary file 1 — Supporting Information [file VRO2-8-e14-s001.pdf]

Supplement 1

Table S1: Technical and didactical aspects identified by the project team for tool selection.

**Aspects covered by the matrix**	
Technical	- Sustainability and maintenance requirements<br>- Licensing costs<br>- Integration into the existing IT infrastructure and applications<br>- Data storage<br>- User interface (back-end)
Didactical	- Built in feedback options and task types<br>- Support and enabling of (inter-)active learning process<br>- User interface (front-end)

Supplement 2

Table S2: List of national requirements for the veterinary interdisciplinary lectures[1] which was divided into clinical medicine and VPH and then assembled with the international Day One Competences and corresponding underpinning knowledge and understanding for veterinary education[2].

**Domain**	**National requirements, according to §53 TAppV**	**Manual of Standard Operation Procedures, according to EAEVE, FVE 2016**	
		**Day One Competences**	**Underpinning Knowledge and Understanding**
**VPH**	Food safety, Milk hygiene:<br>Risk assessment, Quality management and marketability of food products	Perform ante-mortem inspection of animals destined for the food-chain, including paying attention to welfare aspects; correctly identify conditions affecting the quality and safety of products of animal origin, to exclude those animals whose condition means their products are unsuitable for the food-chain.<br>Perform inspection of food and feed including post-mortem inspection of food producing animals and inspection in the field of food technology.	Veterinary public health issues including […] food hygiene and technology.
	Animal disease control/ Zoonoses<br>➔ Options for painless killing	Recognize suspicious signs of possible notifiable, reportable and zoonotic diseases and take appropriate action, including notifying the relevant authorities.	Awareness of other diseases of international importance that pose a risk to national and international biosecurity and trade.<br>Legislation relating to […] animal movement, notifiable and reportable disease.
	Animal Welfare<br>Ethology	-	The structure, function and behaviour of animals and their physiological and welfare needs, including healthy common domestic animals, captive wildlife and laboratory-housed animals.<br>Legislation relating to animal care and welfare […]
	Residue problems, environmental contaminants and animal hygiene	-	-

	Epidemiology	Advise on, and implement, preventative programs appropriate to the species and in line with accepted animal health, welfare and public health standards.	Veterinary public health issues, including epidemiology, transboundary epizootic diseases, zoonotic and food-borne diseases, emerging and re-emerging diseases […].
**VPH/ Clinical**	Animal husbandry<br><br>herd health management<br><br>Animal/Livestock breeding	-	A knowledge of the businesses related to animal breeding, production and keeping.<br><br>The principles of disease prevention and the promotion of health and welfare.
**Veterinary clinical medicine**	Internal medicine<br><br>Animal Reproduction<br><br>Surgery<br><br>→ Identify and process the origin, diagnosis and treatment of diseases with specific cases<br><br>→ Considerate specially the impact of the use of ionising radiation or radioactive substances	Prepare accurate clinical and client records, and case reports when necessary, in a form satisfactory to colleagues and understandable by the public.<br>Obtain an accurate and relevant history of the individual animal or animal group, and its/their environment.<br>Perform a complete clinical examination and demonstrate ability in clinical decision-making.<br><br>Develop appropriate treatment plans and administer treatment in the interests of the patients and with regard to the resources available.<br><br>Collect, preserve and transport samples, select appropriate diagnostic tests, interpret and understand the limitations of the test results.<br><br>Understand the contribution that imaging and other diagnostic techniques can make in achieving a diagnosis. Use basic imaging equipment and carry out an examination effectively as appropriate to the case, in accordance with good health and safety practice and current regulations.<br><br>Perform aseptic surgery correctly.	The aetiology, pathogenesis, clinical signs, diagnosis and treatment of the common diseases and disorders that occur in all common domestic species.

	Assess and manage pain.<br><br>Recognise when euthanasia is appropriate and perform it with respect of the animal, using an appropriate method, whilst showing sensitivity to the feelings of owners and others, with due regard to the safety of those present; advise on disposal of the carcass.	
Pathological and topographical anatomy	Perform a systematic gross post-mortem examination, record observations, sample tissues, store and transport them.	-
Clinical pharmacology	Access the appropriate sources of data on licensed medicines.<br>Prescribe and dispense medicines correctly and responsibly in accordance with legislation and latest guidance.<br><br>Safely perform sedation, and general and regional anaesthesia; implement chemical methods of restraint.	Medicines legislation and guidelines on responsible use of medicines, including responsible use of antimicrobials and antiparasitic drugs.
Animal nutrition	Assess the physical condition, welfare and nutritional status of an animal or group of animals and advise the client on principles of husbandry and feeding.	-
Veterinary professional legislation	-	-

[1] Bundesministerium der Justiz und für Verbraucherschutz. Verordnung zur Approbation von Tierärztinnen und Tierärzten vom 27. Juli 2006 (BGBl. I S. 1827), die zuletzt durch Artikel 7 des Gesetzes vom 15. August 2019 (BGBl. I S. 1307) geändert worden ist. [Federal Minister of Justice and Consumer Protection. Ordinance on the licensing of veterinarians of July 27, 2006 (Federal Law Gazette I p. 1827), last amended by Article 7 of the Act of August 15, 2019 (Federal Law Gazette I p. 1307)]: TAppV 2006.

[2] European Association of Establishments for Veterinary Education (EAEVE), Federation of Veterinarians of Europe (FVE). European System of Evaluation of Veterinary Training (ESEVT): Manual of Standard Operating Procedures 2016. Available at: https://www.eaeve.org/fileadmin/downloads/SOP/ESEVT_SOP_May_2016_amended_Annex_8_approved_by_ExCom_on_29_May_2019.pdf Accessed May 22, 2020.

Supplement 3

Table S3: List of all cases in the interdisciplinary lectures (clinic in blue, VPH in green) at the Faculty of veterinary medicine at Freie Universität Berlin over the semesters 6,7 and 8. Also the different clinics and institutes are listed who collaborated together while building the cases (the pilot cases are marked with *).

Case count	Case diagnosis	Species	Module	Interdisciplinary Collaboration	Sem.
**1**	Equine Colic - Relocation of the colon ascendens	Equine	Gastrointestinal diseases	Equine Clinic; Institute of Animal Nutrition; Institute of Veterinary Anatomy; Institute of Parasitology and Tropical Veterinary Medicine	6
**2**	Castration of unanaesthetised piglets	Swine	Animal Welfare	Institute of Animal Welfare, Animal Behaviour and Laboratory Animal Science; Institute of Animal Nutrition; Institute of Virology; Institute of Pharmacology and Toxicology; Ruminant and Swine Clinic	
**3**	Illegal puppy trade	Canine			
**4**	Inappropriate animal husbandry	Canine			
**5**	Pregnancy diagnosis with suspected luteal insufficiency*	Canine	Animal Reproduction	Clinic of Animal Reproduction; Institute of Animal Nutrition; Institute of Veterinary Anatomy, Institute of Veterinary Pathology; Small Animal Clinic	7
**6**	Pyometra	Canine			
**7**	Benign prostatic hyperplasia	Canine			
**8**	Atrial fibrillation, Mitral valve insufficiency	Equine	Cardiology	Equine Clinic; Institute of Veterinary Physiology; Institute of Pharmacology and Toxicology; Institute of Veterinary Anatomy	
**9**	Outbreak of *campylobacter**	Human (Zoonosis)	Outbreak investigation and zoonosis	Institute of Food Safety and Food Hygiene; Institute for Veterinary-Epidemiology and Biostatistics; Institute of Microbiology and Epizootics; Clinic of Reproduction	
**10**	*Listeria* outbreak	Sheep (Zoonosis)			
**11**	*Vibrio* outbreak	Human (Zoonosis)			
**12**	*Salmonella* outbreak	Human (Zoonosis)			
**13**	Lid-edge tearing	Equine	Ophthalmology	Small Animal Clinic – Ophthalmology and Internal Medicine; Institute of Veterinary Anatomy, Institute of Microbiology and Epizootics	8
**14**	Hypertensive Retinopathy, Hypertension and chronic kidney insufficiency	Feline			

**15**	Uveitis, Glaucoma, Feline infectious peritonitis	Feline		
**16**	Equine recurrent uveitis, Leptospirosis	Equine		
**17**	Cataract, diabetes mellitus	Canine		
**18**	Equine Asthma, laryngeal paralysis	Equine	Complex diseases	Equine Clinic; Institute of Pharmacology and Toxicology; Institute of Veterinary Anatomy; Institute of Animal Nutrition
**19**	Laminitis, Endocrinopathies	Equine		
**20**	Anthrax outbreak	Ruminant	Epizootic outbreak	Institute of Microbiology and Epizootics; Institute of Food Safety and Food Hygiene; Institute for Veterinary-Epidemiology and Biostatistics; Institute for Animal Hygiene and Environmental Health; Institute of Pathology, Institute of Veterinary Biochemistry
**21**	American Foulbrood outbreak	Bees		
**22**	Outbreak of pseudotuberculosis	Ruminant		
**23**	*Salmonella* outbreak	Galliformis		
